# Gene Expression Analysis of Zebrafish Heart Regeneration

**DOI:** 10.1371/journal.pbio.0040260

**Published:** 2006-08-01

**Authors:** Ching-Ling Lien, Michael Schebesta, Shinji Makino, Gerhard J Weber, Mark T Keating

**Affiliations:** **1**Department of Cardiology, Children's Hospital, Boston, Massachusetts, United States of America; **2**Department of Cell Biology, Harvard Medical School, Boston, Massachusetts, United States of America; **3**Children's Hospital Stem Cell Program, Department of Hematology/Oncology, Howard Hughes Medical Institute, Harvard Medical School, Boston, Massachusetts, United States of America; Wellcome Trust Sanger InstituteUnited Kingdom

## Abstract

Mammalian hearts cannot regenerate. In contrast, zebrafish hearts regenerate even when up to 20% of the ventricle is amputated. The mechanism of zebrafish heart regeneration is not understood. To systematically characterize this process at the molecular level, we generated transcriptional profiles of zebrafish cardiac regeneration by microarray analyses. Distinct gene clusters were identified based on temporal expression patterns. Genes coding for wound response/inflammatory factors, secreted molecules, and matrix metalloproteinases are expressed in regenerating heart in sequential patterns. Comparisons of gene expression profiles between heart and fin regeneration revealed a set of regeneration core molecules as well as tissue-specific factors. The expression patterns of several secreted molecules around the wound suggest that they play important roles in heart regeneration. We found that both *platelet-derived growth factor-a* and *-b (pdgf-a* and *pdgf-b)* are upregulated in regenerating zebrafish hearts. PDGF-B homodimers induce DNA synthesis in adult zebrafish cardiomyocytes. In addition, we demonstrate that a chemical inhibitor of PDGF receptor decreases DNA synthesis of cardiomyocytes both in vitro and in vivo during regeneration. Our data indicate that zebrafish heart regeneration is associated with sequentially upregulated wound healing genes and growth factors and suggest that PDGF signaling is required.

## Abstract 2

Comparison of gene expression in regenerating heart and fin points to common as well as tissue-specific mechanisms. PDGFs expressed in the heart induce DNA synthesis in cardiac myocytes.

## Introduction

Injured mammalian hearts cannot regenerate; instead, they scar. Mammalian cardiomyocytes undergo hypertrophy to compensate for the loss of cardiac cells. Although it has been reported that cardiomyocytes in diseased human hearts can proliferate [ 1], most evidence suggests that this is not a major response after heart injury [ 2]. Some reports have suggested that bone marrow stem cells and cardiac stem cells may play a role in cardiac regeneration in humans, but this remains controversial [ 3– 5]. By contrast with mammals, newt and zebrafish hearts regenerate after amputation [ 6, 7]. The molecular mechanisms underlying this phenomenon have not been characterized in newts because of a lack of genetic tools. Recently, we [ 8] and others [ 9] showed that zebrafish fully regenerate myocardium after 20% ventricular resection. Thus, the zebrafish provides a genetically tractable model system in which to study the molecular mechanisms of heart regeneration.

Zebrafish heart regeneration occurs over a 2-mo period [ 8]. After amputation, a blood clot forms to seal the ventricle and stop bleeding. The blood clot is replaced by a fibrin clot at 2 to 3 d postamputation (dpa). The regenerating myocardium is derived from cardiomyocytes surrounding the wound that reenter the cell cycle, presumably in response to signals from the wound [ 10]. Cardiomyocytes initiate DNA synthesis and proliferation at 7 dpa. Between 7 and 14 dpa, DNA synthesis and proliferation of cardiomyocytes reach a peak [ 8]. Nascent cardiomyocytes replace most of the lost ventricular tissue by 30 dpa, and the structure of the heart is fully restored at 60 dpa [ 8].

Elucidating the molecular mechanism of zebrafish heart regeneration may provide insight into potential therapeutic approaches for heart injury in humans. In an effort to identify genes important for heart regeneration, we tested whether zebrafish mutants defective in fin regeneration also have heart regeneration defects. Two fin regeneration mutants, *ncp* [ 8, 11] and *nbl* [ 12], are also defective for heart regeneration. *ncp* encodes the zebrafish *monopolar spindle (mps) 1* gene that is involved in mitotic checkpoint regulation [ 11]. *nbl* encodes the heat shock protein 60 (hsp60) chaperone protein [ 12]. Using a candidate gene approach, Raya et al. [ 9] have identified a limited set of genes that are expressed during heart regeneration. Collectively, these studies reveal little information on the initiation and overall progression of heart regeneration, prompting us to take a more systematic approach.

In order to identify genes that are important for zebrafish heart regeneration, we utilized microarray technology to carry out gene expression profiling of regenerating hearts at 3, 7, and 14 dpa. Distinct gene clusters were identified according to their temporal expression patterns and were classified into different functional categories. Expression of genes encoding for wound response/inflammatory factors, secreted molecules, and matrix metalloproteinases (MMPs) increased in sequential patterns. Comparisons of gene expression profiles between regenerating hearts and fins suggest that these two processes share common molecules but also use tissue-specific factors. To identify signals that trigger regeneration, we focused our analysis on secreted molecules. *platelet-derived growth factor-a (pdgf-a)* and several other secreted molecules, including the previously unidentified *pdgf-b,* were upregulated in regenerating hearts. In primary cultures of adult zebrafish cardiomyocytes, PDGF-B homodimers induce DNA synthesis. In addition, treatment with a chemical inhibitor of PDGF receptor resulted in a decrease in DNA synthesis in vitro and in regenerating hearts in vivo. These data suggest that PDGF signaling is required for zebrafish heart regeneration and show that microarray analysis is a valuable approach to study the molecular mechanisms of this process.

## Results

### Gene Expression Profiling of Zebrafish Heart Regeneration

To identify molecular signals that initiate regeneration, we focused our gene expression profile analysis on the early stages of zebrafish heart regeneration. Sham-operated hearts and regenerating hearts were collected at 3, 7, and 14 dpa. To enrich for transcripts that are involved in regeneration, we dissected one third of the ventricle containing the amputation plane. Ten to 12 heart regenerates were dissected and pooled for analysis.

**Figure 1:**
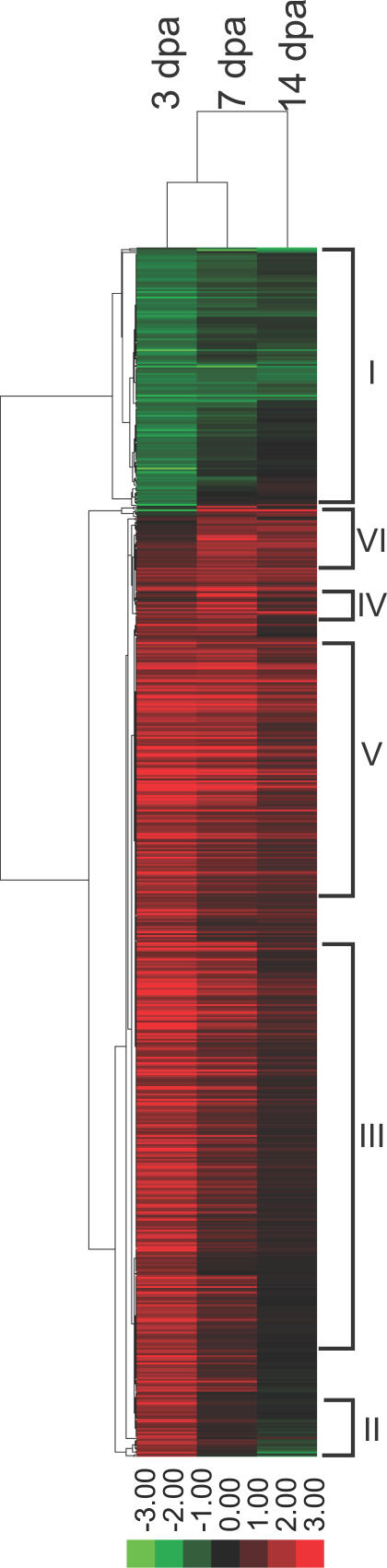
Gene Expression Profiling of Zebrafish Heart Regeneration A total of 662 genes are differentially expressed during zebrafish heart regeneration. These genes were clustered into groups using the hierarchical method based on the similarity of their expression patterns during heart regeneration at 3, 7, and 14 dpa. The color chart indicating fold change of expression uses a base 2-logarithm scale. Red and green represent increased and decreased expression, respectively. Pairwise comparisons revealed that regenerating hearts at 3 dpa had the most differentially expressed genes and that 3 dpa and 7 dpa regenerating hearts shared a high percentage of genes that had increased expression during regeneration.

We used Affymetrix zebrafish GeneChips to perform transcriptional profiling. This allowed simultaneous analysis of approximately 14,900 transcripts. According to the annotation of the transcripts at the time of manuscript preparation, these represent 10,318 genes, providing approximately 45% coverage of the zebrafish genome. We identified 662 transcripts that are differentially expressed in at least one of the three time points tested during the regeneration process ( Dataset S1). Hierarchical clustering revealed groups of genes that were expressed at different time points during regeneration ( Figures 1 and S1). Pairwise comparisons revealed that regenerating hearts at 3 dpa had the highest number of differentially expressed genes and that regenerating hearts at 3 dpa and 7 dpa shared a high percentage of genes with increased expression ( Figure 1).

Expression of 142 genes (21.4%; Figure S1, cluster I) were generally decreased during the regeneration process; 53 (8.0%) genes were specifically expressed at 3 dpa ( Figure S1, cluster II); 232 genes (35.0%) were preferentially expressed at 3 and 7 dpa ( Figure S1, cluster III); 26 genes (3.9%) were most highly expressed at 7 dpa ( Figure S1, cluster IV); 180 genes (27.1%) were expressed across the early regeneration process ( Figure S1, cluster V); and 32 genes (4.8%) were mainly expressed at 7 to 14 dpa ( Figure S1, cluster VI). Representative genes in each cluster are illustrated in Figure S1. *mps1,* which we have shown to be required for heart regeneration by genetic analysis [ 8], was identified in cluster II. In summary, microarray analysis of zebrafish heart regeneration has revealed a set of genes that likely regulate multiple steps of the initial stages of this regeneration process.

### Zebrafish Heart Regeneration Involves Wound Healing, Expression of Secreted Molecules, and Tissue Remodeling

**Figure 2:**
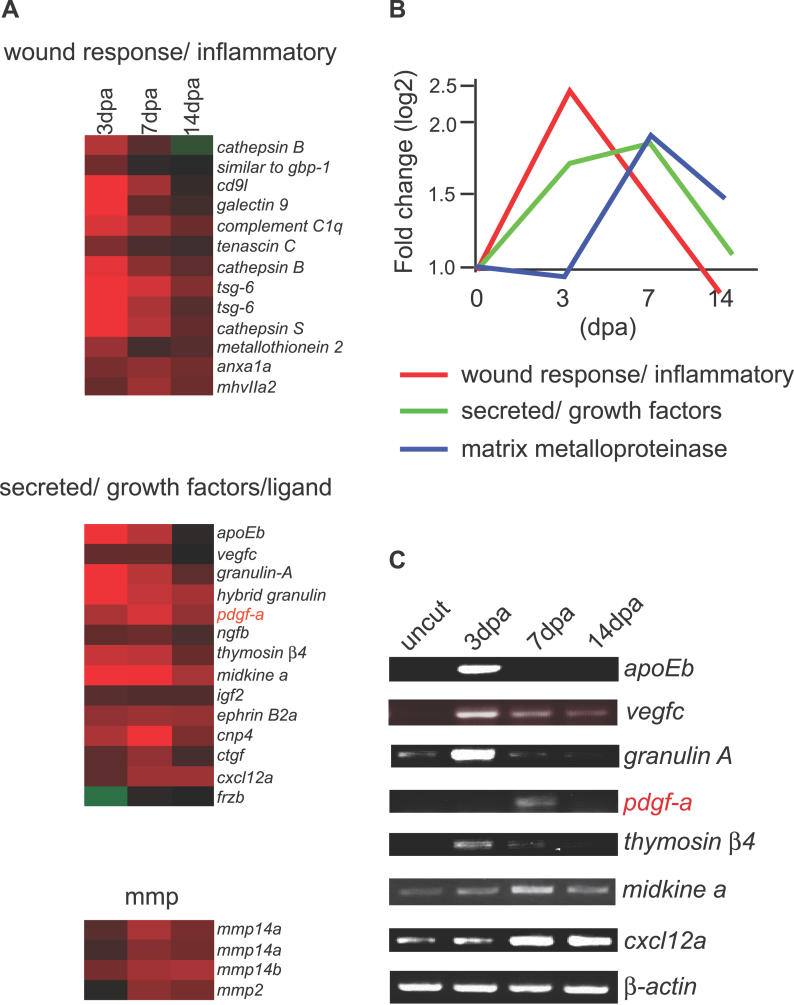
Wound Response/Inflammatory Factors, Secreted Molecules, and MMPs Are Upregulated during Zebrafish Heart Regeneration (A) Genes encoding wound response and inflammatory factors, secreted molecules, and MMPs were clustered based on their expression patterns at 3, 7, and 14 dpa. The gene name, gene symbol, and fold change of each gene can be found in Dataset S1. The color chart indicates fold change of expression, and red and green represent increased and decreased expression, respectively. (B) General trend and average fold change of expression of wound response/inflammatory genes, secreted molecules, and MMPs. The wound response/inflammatory genes are expressed early (peak at 3 dpa) during zebrafish heart regeneration. Genes coding for secreted molecules begin to express at 3 dpa and the expression level reach a peak at 7 dpa. The MMPs start to express at 7 dpa and last until 14 dpa. (C) Semiquantitative RT-PCR of a subset of genes coding for secreted molecules, including *apoEb (apolipoprotein Eb), vegfc (vascular endothelial growth factor c), granulin A, pdgf-a (platelet-derived growth factor-a;* labeled in red) *, thymosin* β *4, midkine a*, *and cxcl12a (chemokine (c-x-c motif) ligand 12a)*. β *-actin* was used as a loading control. The RT-PCR results confirmed the temporal expression patterns of these genes as determined by microarray analysis.

We clustered these 662 differentially expressed genes according to different functional categories and expression patterns ( Dataset S1). We found that genes coding for wound response/inflammatory/anti-inflammatory factors, secreted molecules, and MMPs are expressed in sequential patterns ( Figure 2A and 2B). The wound response/inflammatory genes were expressed early during zebrafish heart regeneration, peaking at 3 dpa ( Figure 2A and 2B). The expression of these genes most likely reflects the recruitment of inflammatory cells to the wound site after the injury. Expression of a set of secreted molecules began at 3 dpa and expression levels reached a peak at 7 dpa ( Figure 2A and 2B). Several genes encoding growth factors/secreted molecules, such as *apoEb, vegf-c,* and *granulin-A,* were strongly expressed at 3 dpa ( Figure 2A and 2C) and are likely involved in the wound healing process. *vegf-c* is required for vasculogenesis and angiogenesis in the zebrafish embryo [ 13] and may play a role in the wound healing response to heart injury and regeneration. *granulin A,* a growth factor gene involved in wound response [ 14], was also strongly upregulated at 3 dpa ( Figure 2A and 2C). Expression of the genes coding for MMP2, MMP14a, and MMP14b *(mmp2, mmp14a,* and *mmp14b)* ( Figure 2A and 2C) and acidic cysteine–rich glycoprotein *(sparc)* ( Figure S1) began at 7 dpa and lasted until 14 dpa. These molecules are involved in the regulation of the extracellular matrix [ 15, 16], suggesting that extensive tissue remodeling occurs at later stages of heart regeneration.

### Heart Regeneration and Fin Regeneration Use Both Common and Tissue-Specific Factors

To determine whether zebrafish heart and fin regeneration share common mechanisms, we compared the gene expression profiles between these two processes. Of 662 transcripts that are differentially expressed during heart regeneration, 132 genes were also differentially expressed during fin regeneration ( Table S1 and [ 17]). One hundred nineteen transcripts were upregulated and 13 transcripts were downregulated during both heart and fin regeneration. *mps1* was one of the commonly upregulated genes. This is consistent with our previous findings that *ncp* mutants are defective for both heart and fin regeneration [ 8, 11]. Among these 132 genes, transcripts coding for extracellular matrix and cell adhesion proteins represented the most differentially expressed genes, suggesting that tissue remodeling and cell migration play important roles in both heart and fin regeneration. *midkine a,* which encodes a heparin binding growth factor [ 18], is upregulated in both heart and fin regeneration, suggesting it is important for both processes. However, there were 491 transcripts that were differentially expressed only during heart regeneration but not significantly changed during fin regeneration, and more than 600 genes that were differentially expressed during fin regeneration [ 17] but not significantly changed during heart regeneration. In addition, 11 transcripts showed opposite expression patterns between fin and heart regeneration ( Table S1). Collectively, these data indicate that a set of core molecules are differentially regulated in both zebrafish heart and fin regeneration but that many tissue-specific factors are also required.

### Secreted Molecules and Growth Factors Are Differentially Expressed in Regenerating Zebrafish Heart

What are the signals that initiate heart regeneration, and where do they originate? Most proliferating cardiomyocytes are localized to the lateral sides of the wound [ 8]. Thus, it is likely that initiation signals for postinjury cardiomyocyte proliferation are secreted molecules from the wound [ 10]. We therefore further analyzed several genes coding for growth factors/secreted molecules. *apoEb, vegfc, thymosin* β *4, granulin A, pdgf-a, midkine a, insulin-like growth factor 2 (igf2),* and *chemokine c-x-c ligand 12 (cxcl12)* were upregulated during the early stages of regeneration ( Figures S1 and 2A). The temporal expression patterns of these secreted factors as determined by microarray analysis were independently confirmed by semiquantitative RT-PCR ( Figure 2C).

*apoEb, vegfc,* and *granulin A* are likely to be involved in the wound-healing process because they were strongly expressed at 3 dpa, and expression dramatically decreased afterward. *igf2* has been previously shown to induce DNA synthesis in fetal rat cardiomyocytes and hypertrophy in neonatal rat cardiomyocytes [ 19]. *thymosin* β *4,* which encodes a G-actin sequestering peptide that is secreted into the wound fluid and promotes cardiac cell migration, survival, and repair [ 20, 21], was upregulated at both 3 and 7 dpa ( Figures S1 and 2). *midkine a* was upregulated as early as 3 dpa and its expression lasted until 14 dpa ( Figures S1 and 2). *pdgf-a* was upregulated at 7 dpa. Finally, *cxcl12,* which is involved in stem cell mobilization [ 22], was upregulated at 7 and 14 dpa ( Figures S1 and 2).

**Figure 3:**
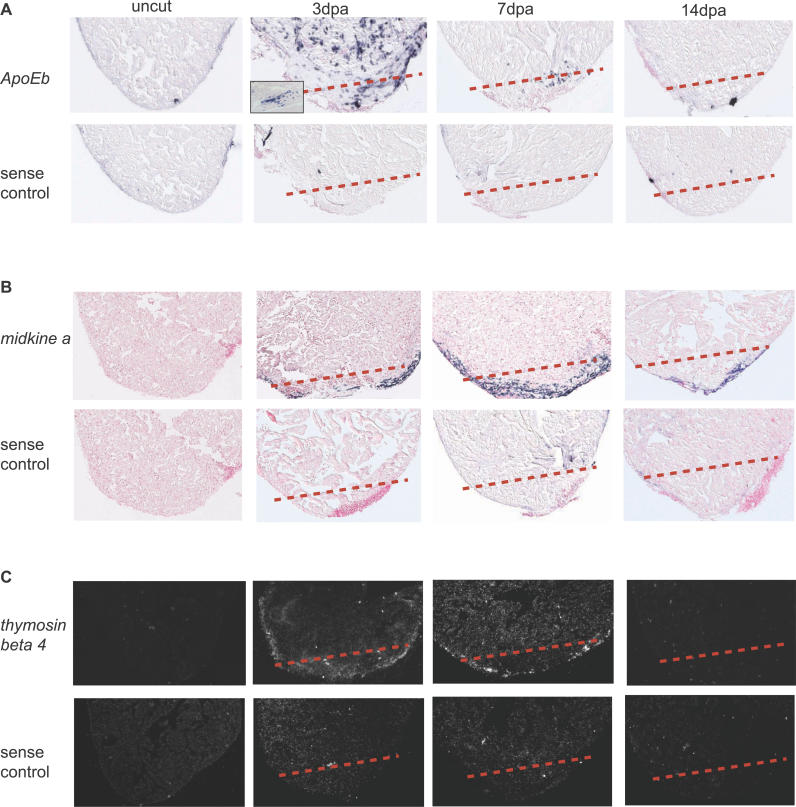
apoEb, midkine a, and *thymosin* β *4* Are Expressed around the Wound Site during Zebrafish Heart Regeneration Spatial expression patterns of *apoEb*, *midkine a,* and *thymosin* β *4* in sham-operated hearts (uncut) and regenerating hearts at 3, 7, and 14 dpa were determined by in situ hybridization. (A) The expression pattern of *apoEb* was determined using a DIG-labeled antisense probe. *apoEb* was highly expressed at 3 dpa; its punctate expression suggests that it is expressed by infiltrating macrophages (see the higher magnification image in the inset). (B) Expression level of *midkine a* was upregulated from 3 dpa, reaches a peak at 7 dpa and lasts until 14 dpa. It appears to be expressed around the wound site in the compact layer of myocardium and the epicardium. (C) The expression pattern of *thymosin* β *4* was determined using a radioactive antisense probe. *thymosin* β *4* appears to be expressed around the wound and surrounding compact myocardium. Sense probes for each gene were used as negative controls. A dashed line marks the amputation plane.

We examined the spatial expression patterns of some of these genes by in situ hybridization. *apoEb* was strongly expressed in the regenerating heart at 3 dpa and was not expressed at all in the uninjured heart ( Figure 3A). Interestingly, *apoEb* expression was detected around the wound and also inside the trabecular myocardium. The spotted expression pattern suggests that the cells expressing *apoEb* might be infiltrating macrophages ( Figure 3A). Similar expression patterns of *apoEb* by infiltrating macrophages were also reported for regenerating fin [ 23]. We also determined the expression pattern of *midkine a*. Consistent with the microarray and RT-PCR data, *midkine a* expression in the regenerating heart started at 3 dpa, peaked at 7 dpa, and lasted to 14 dpa ( Figure 3B). *midkine a* expression was localized to the wound and the epicardium surrounding the wound. *thymosin* β *4* was detected in regenerating hearts at 3 and 7 dpa but not at 14 dpa or in uninjured hearts; its expression was localized to the wound and the surrounding compact myocardium ( Figure 3C). Thus, the growth factors/secreted molecules encoded by these differentially expressed genes may be involved in the initiation of different aspects of heart regeneration.

### *pdgf-a* and - *b* Are Upregulated in Regenerating Zebrafish Heart

*pdgf-a* expression was upregulated at 7 dpa during heart regeneration ( Figure 2A and 2C). The classic PDGFs, PDGF-A and PDGF-B, can form homodimers and heterodimers and bind to their tyrosine kinase receptors, PDGFRα and PDGFRβ [ 24]. The zebrafish *pdgf-b* gene was not present on the Affymetrix genome array and had not been previously cloned. As PDGF-A can form heterodimers with PDGF-B, we cloned and characterized a *pdgf-b* partial cDNA from regenerating zebrafish heart using RT-PCR. The predicted amino acid sequence of zebrafish PDGF-B is highly homologous to human, mouse, and *Xenopus* PDGF-B ( Figure S2).

**Figure 4:**
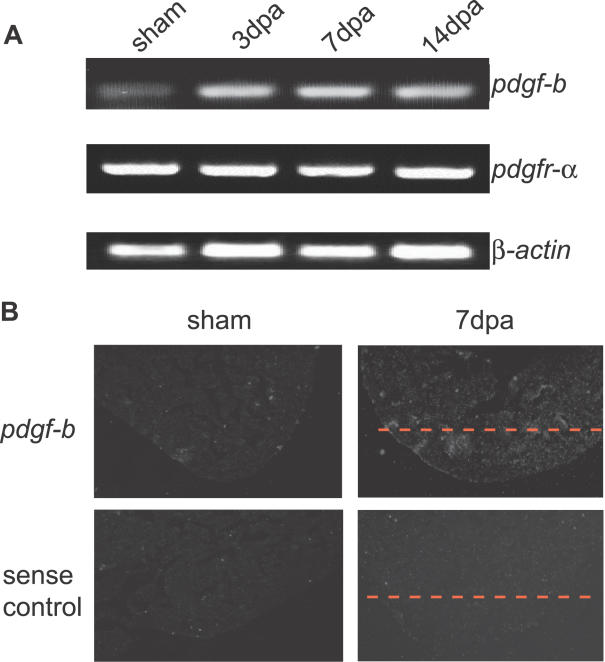
pdgf-b Expression Is Upregulated in Regenerating Zebrafish Hearts (A) RT-PCR analysis of *pdgf-b* and *pdgfr-*α. β *-actin* was used as a loading control. Expression of *pdgf-b* began to increase at 3 dpa and lasted until 14 dpa during zebrafish heart regeneration. *pdgfr-*α was expressed in both sham-operated and regenerating hearts; its expression level does not change. (B) In situ hybridization using a radioactive antisense probe showing *pdgf-b* expression in sham-operated and regenerating hearts at 7 dpa. Expression *of pdgf-b* was localized to the wound site. A radioactive sense probe was used as a negative control. The dashed red line marks the amputation plane.

Semiquantitative RT-PCR revealed that *pdgf-b* was also upregulated during the regeneration process ( Figure 4A and 4B). To determine the expression pattern of *pdgf-b,* we performed radioactive in situ hybridization. As shown in Figure 4B, *pdgf-b* appeared to be expressed in the regenerating heart around the wound. PDGFRα is the common receptor for PDGF-AA, PDGF-AB, and PDGF-BB homodimers and heterodimers. In contrast to *pdgf-a* and - *b,* microarray and RT-PCR analysis showed that *pdgfr*α was expressed in the noninjured zebrafish hearts at low levels and that expression levels did not change during regeneration ( Figure 4A). The upregulation of *pdgf-a* and - *b* at 7 dpa coincides with the time point in which DNA replication starts to increase in cardiomyocytes. Thus, PDGF signaling may play a critical role in initiating cardiomyocyte proliferation during zebrafish heart regeneration.

### PDGF-BB Induces DNA Synthesis in Cardiomyocytes

**Figure 5:**
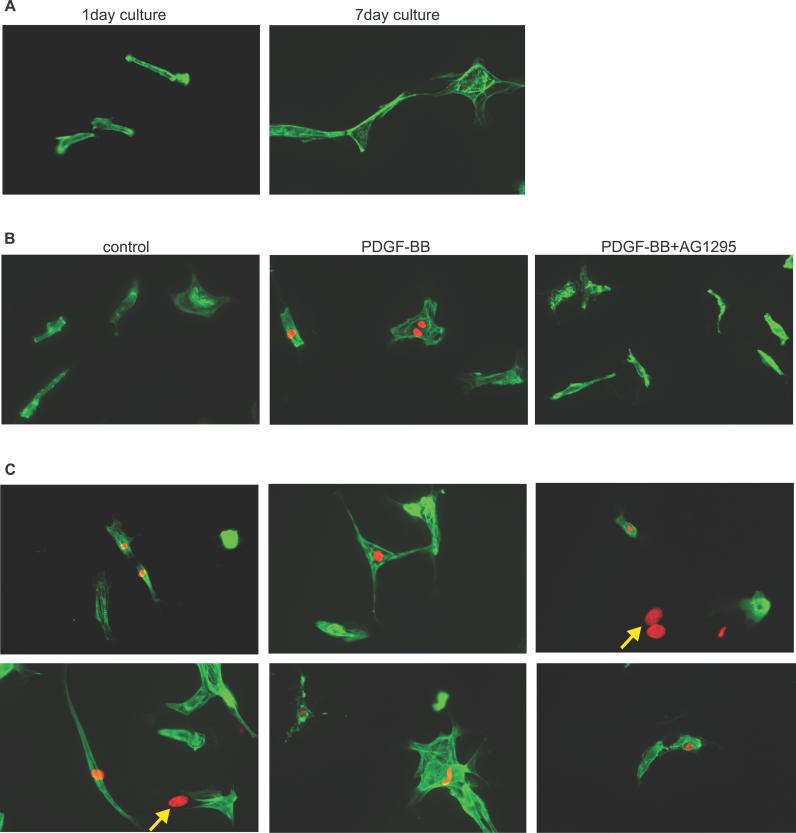
PDGF-B Induces DNA Synthesis in Adult Zebrafish Cardiomyocytes (A) Adult zebrafish cardiomyocytes were isolated and cultured for 1 or 7 d. The cardiomyocyte population is heterogeneous in size and shape consisting mostly of rod-shape cells. To confirm that the cells are cardiomyocytes, the cells were stained with anti-tropomyosin antibody (green) and anti-MEF2 antibody (unpublished data). After 7 d in culture in 10% FBS, the cardiomyocytes underwent dedifferentiation; the cells lose their rod shape and striation becomes disorganized. (B) DNA synthesis as determined by BrdU incorporation in adult zebrafish cardiomyocytes treated with DMSO (control), PDGF-BB, and PDGF-BB plus the PDGF receptor inhibitor, AG1295. BrdU labeling is shown in red and tropomyosin staining is shown in green. PDGF-BB induced a 2.68-fold increase in DNA synthesis compared to control in adult zebrafish cardiomyocytes ( *p* < 0.05). The effect of PDGF-BB inducing DNA synthesis can be blocked by AG1295. (C) PDGF-BB induced a variety of cardiomyocytes with different size and shape to undergo DNA synthesis. Noncardiomyocytes are marked by yellow arrows.

Zebrafish hearts regenerate by cardiomyocyte proliferation. Factors secreted from the wound might induce proliferation of surrounding cardiomyocytes. To determine the functions of the secreted factors identified by gene expression analysis at the cellular level, we turned to cultured cardiomyocytes. We modified a previously published protocol for primary culture of zebrafish adult cardiomyocytes [ 25] to obtain a larger quantity of cells that can be cultured for 7 to 10 d. Cardiomyocytes in our primary culture were positively identified by staining with antibodies against the cardiomyocyte markers tropomyosin ( Figure 5A) and MEF2 (unpublished data). In culture, the cardiomyocytes gradually underwent dedifferentiation, characterized by disorganized cytoskeletal striation and loss of its rod-shaped morphology ( Figure 5A), similar to the previously reported phenomena for adult rat and newt cardiomyocytes in culture [ 26, 27]. We tested the effects of apoE, Granulin, midkine, and PDGF-AA, -AB, and -BB homodimers and heterodimers in inducing DNA synthesis on zebrafish cardiomyocytes ( Table S2). Among these factors, we observed that PDGF-BB increased BrdU incorporation (2.68 ± 0.05 fold, *p* < 0.05) compared to control after 7 d of treatment ( Figure 5B) while the other factors tested had little or no effects ( Table S2). We did not detect mitosis in PDGF-BB treated cardiomyocytes by staining with anti-phosphorylated histone-3 antibody (unpublished data). To determine whether mammalian PDGF-BB and the zebrafish homolog have different effects, we produced recombinant zebrafish PDGF-BB protein (zPDGF-BB). zPDGF-BB induced similar levels of DNA synthesis as mammalian PDGF-BB in zebrafish cardiomyocytes but not mitosis (unpublished data). Both PDGF-BB and zPDGF-BB induced DNA synthesis in cardiomyocytes of a variety of different sizes and shapes, indicating that the proliferative capacity of cardiomyocytes is not limited to a specific subpopulation of cardiomyocytes ( Figure 5C). To demonstrate that PDGF treatment on zebrafish cardiomyocytes is specifically activating the PDGF signaling pathway, we utilized a selective PDGFR chemical inhibitor, AG1295 [ 28]. AG1295 selectively inhibits PDGFR tyrosine kinase activity [ 29, 30] and inhibits PDGF signaling required for morphogenic movement during zebrafish gastrulation [ 31]. In cultured adult zebrafish cardiomyocytes, AG1295 inhibited the DNA synthesis induced by PDGF-BB ( Figure 5B). We conclude that PDGF signaling induces DNA synthesis in zebrafish cardiomyocytes in vitro.

### PDGF Signaling Is Required for Cardiomyocyte Proliferation during Zebrafish Heart Regeneration

**Figure 6:**
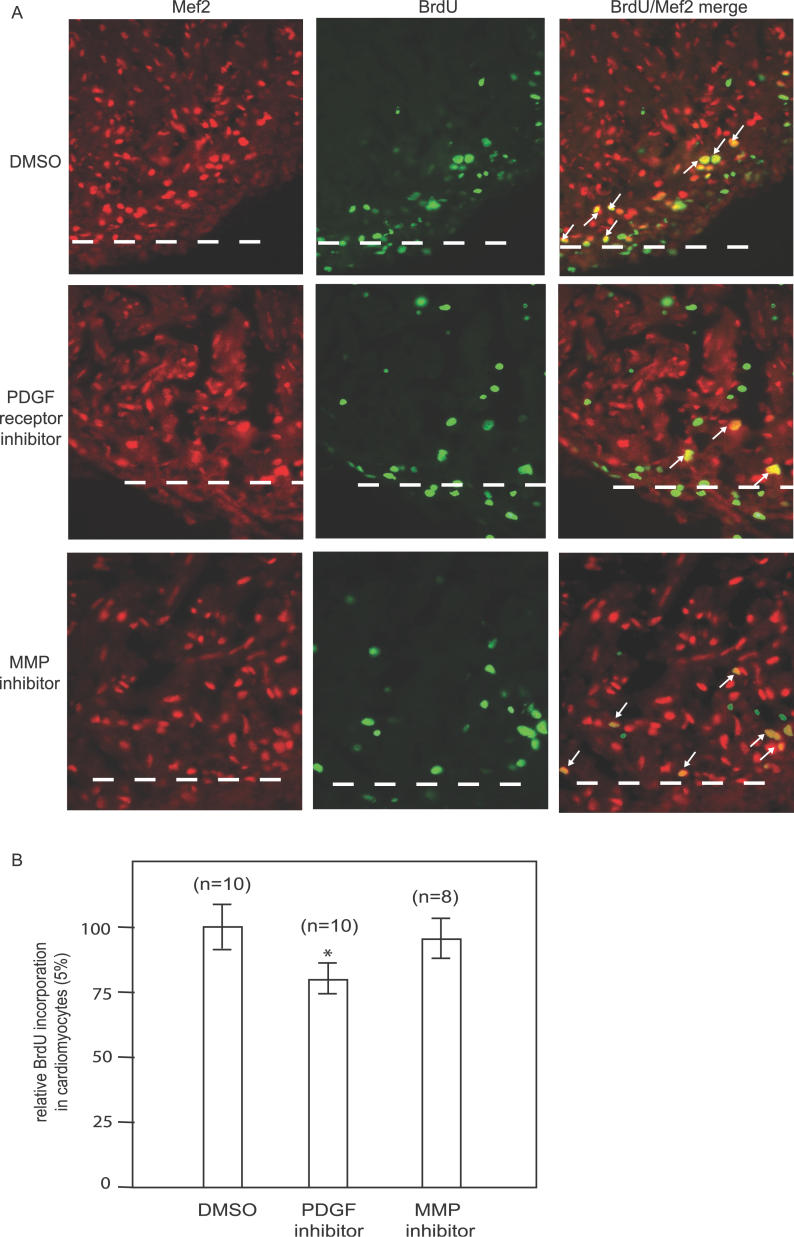
PDGF Signaling Is Required for Cardiomyocyte Proliferation during Zebrafish Heart Regeneration After heart surgery, the fish were allowed to recover for one day and were then treated with either the PDGF receptor inhibitor AG1295, DMSO as a negative control, or the MMP inhibitor GM6001 as a specificity control. (A) Heart sections were stained with MEF2 antibody (red) to visualize the cardiomyocyte nuclei and with BrdU antibody (green) to measure DNA synthesis. The BrdU/MEF2 double-positive nuclei (white arrows) are shown in yellow. Many BrdU-positive cells are not cardiomyocytes. PDGF receptor inhibitor treatment resulted in fewer BrdU-positive cardiomyocytes while DMSO and MMP inhibitor treatment did not. The dashed white line marks the amputation plane. (B) Quantification of DNA synthesis in cardiomyocytes during zebrafish heart regeneration. Results were normalized to DMSO-treated control hearts (100%). AG1295 treated hearts had 16% ( *p* < 0.05) fewer BrdU-positive cardiomyocytes compared to DMSO control hearts (Student's *t*-test), whereas the difference between GM6001-treated and DMSO-treated hearts was statistically insignificant ( *p* > 0.05). Error bars indicate standard error of the mean.

To address the role of PDGF signaling in zebrafish heart regeneration in vivo, we tested the effect of AG1295 on DNA synthesis in cardiomyocytes. After heart surgery, the fish were allowed to recover for 1 d and were treated with AG1295 or DMSO as a negative control from 2 dpa to 14 dpa. To rule out potential nonspecific effects caused by treatment with chemical inhibitors, we used another chemical inhibitor as a specificity control. From our microarray analysis, we found that the genes coding for the metalloproteinases MMP2 and MMP14 were upregulated at 7 to 14 dpa. We tested whether the MMP inhibitor GM6001 [ 32] would inhibit DNA synthesis of cardiomyocytes. At 14 dpa, fish treated with DMSO and inhibitors were sacrificed to assess DNA synthesis in cardiomyocytes. While cardiomyocytes of zebrafish treated with DMSO ( *n* = 10), GM6001 ( *n* = 8), and AG1295 ( *n* = 10) still underwent DNA synthesis ( Figure 6A), cardiomyocytes from AG1295-treated fish had 16% fewer BrdU-positive cells relative to the DMSO-treated control fish, as determined by BrdU and MEF2 double staining ( *p* < 0.05). By contrast, cardiomyocytes from GM6001-treated fish did not have a significantly reduced level of DNA synthesis compared to DMSO-treated control fish ( *p* > 0.05). Importantly, heart regeneration was inhibited in GM6001-treated fish when assessed at 30 dpa (unpublished data), indicating that GM6001 was efficacious under our assay conditions. These results suggest that PDGF signaling is specifically required for proliferation of a portion of cardiomyocytes in vivo during heart regeneration.

## Discussion

In this study, we have systematically examined gene expression involved in zebrafish heart regeneration using DNA microarray analysis. Transcriptional profiling of regenerating zebrafish hearts revealed genes that are differentially expressed at 3, 7, and 14 dpa during regeneration. The stage-specific regulation suggests that the regeneration process begins with wound healing, followed by increased expression of several genes encoding secreted molecules and then followed by tissue remodeling. Several of these genes were specifically upregulated around the wound site in regenerating hearts and encode secreted molecules that might function as initiation signals for regeneration. Among these was *pdgf-b,* a previously unidentified zebrafish gene. We further showed that PDGF-B signaling contributes to heart regeneration by inducing DNA synthesis of zebrafish cardiomyocytes. Our results demonstrate that microarray analysis is a powerful approach to study heart regeneration, a phenomenon for which forward genetic approaches may not be practical. With a set of candidate genes identified from our microarray analysis, we now can use reverse genetic and pharmacological approaches to study the functions of these genes.

Zebrafish hearts regenerate by cardiomyocyte proliferation around the wound. The signals that trigger regeneration are presumably secreted molecules originating from the wound. Among the genes we identified encoding secreted molecules, several may serve as potential factors that trigger regeneration. For example, the mouse homolog of *thymosin* β *4* has been shown to be protective to the heart after infarction by activating integrin-linked kinase and AKT signaling to promote cardiac cell migration and survival [ 20]. In this study we showed that PDGF-BB homodimers increased DNA synthesis in adult zebrafish cardiomyocytes and that PDGF signaling is required for proliferation of cardiomyocytes during zebrafish heart regeneration. Consistent with our findings, PDGF-BB induces DNA synthesis in adult newt cardiomyocytes [ 33]. We also observed that PDGF-BB induces DNA synthesis in rat neonatal cardiomyocytes (unpublished data), suggesting an evolutionarily conserved mechanism for PDGF signaling in the heart. Hsieh et al. [ 34] recently showed that PDGF-BB delivered into the heart by peptide nanofibers protects against cardiac infarction in rats. The identification of *pdgf-b* and *thymosin* β *4* in our study validates our microarray approach as a strategy for the discovery of genes that may be therapeutic targets for regenerative medicine. The in vivo mechanisms of the PDGF pathway and other factors identified from our microarray analysis in zebrafish heart regeneration remain to be elucidated. Inducible transgenic zebrafish expressing dominant-negative forms of these factors can be used to address these questions via a loss-of-function genetic approach, but this is beyond the scope of this study.

Using a selective inhibitor of PDGF receptor kinase activity, we showed that blocking PDGF signaling results in a decrease of DNA synthesis of cardiomyocytes during heart regeneration. However, DNA synthesis in cardiomyocytes is not completely blocked by inhibition of PDGF signaling. This suggests that the PDGF pathway, although sufficient to induce DNA synthesis in cardiomyocytes, might act in parallel with other signaling pathways to induce cardiomyocyte proliferation and myocardial regeneration. This is supported by our finding that multiple growth factors are upregulated during heart regeneration. For instance, *igf-2* was upregulated during zebrafish heart regeneration. *igf-2* was previously shown to induce DNA synthesis in fetal rat cardiomyocytes [ 19]. In addition, we previously showed that FGF1 induces high level of DNA synthesis in rat cardiomyocytes [ 35]. It is likely that FGF signaling also plays important roles in regulating cardiomyocyte proliferation during heart regeneration. Raya et al. [ 9] reported that *notch1b* and *deltaC* are upregulated during zebrafish heart regeneration, suggesting that the Delta-Notch signaling pathway might be also involved in the control of cardiomyocyte proliferation. Interestingly, *notch1b* and *deltaC* are expressed throughout the ventricle but are excluded from the wound site [ 9]. Thus, we did not detect *notch1b* and *deltaC* upregulation in our microarray analysis.

Comparison of gene expression profiles between fin and heart regeneration revealed a set of common factors as well as tissue-specific factors. Only 20% of genes that were differentially expressed during heart regeneration were also differentially expressed in regenerating fins. This is not surprising, considering that zebrafish hearts regenerate by cardiomyocyte proliferation [ 8], whereas fin regeneration involves the formation of a blastema, a mass of proliferative, pluripotent progenitor cells [ 36]. Furthermore, heart and fin are composed of very different cell types. Thus, many factors involved in the regeneration of these organs must be tissue specific. However, besides the differentially expressed genes in heart and fin regeneration identified by microarray analysis, other genes could be involved in both processes but not regulated at the RNA level. For instance, we previously showed that *hsp60* is required for both fin and heart regeneration [ 12]. *hsp60* is differentially expressed during fin regeneration [ 12, 17], but not during heart regeneration, suggesting that *hsp60* is regulated posttranscriptionally in the heart. Transcripts differentially expressed during both heart and fin regeneration could be involved in other regeneration processes. For instance, *midkine* was previously shown to be important for liver regeneration [ 37]. We found that many common factors are involved in regulation of inflammation, cell adhesion, and the extracellular matrix ( Table S1). For example, MMPs have been shown to be involved in fin regeneration [ 38]. Interestingly, they are also required for newt limb regeneration [ 39]. Extracellular matrix proteins such as fibronectin are upregulated in both heart and fin regeneration ( Table S1). Interestingly, fibronectin is also important for liver regeneration [ 40].

Using the genetically tractable zebrafish as a model organism combined with cDNA microarray technology, we hope to elucidate the molecular mechanisms underlying heart regeneration in lower vertebrates. We have identified genes that serve as regulators during heart regeneration. Together with evolutionarily conserved genetic program among vertebrates, these genes represent promising candidates for future therapeutic approaches in regenerative medicine.

## Materials and Methods

### Zebrafish husbandry, heart amputation, and inhibitors' treatment.

Zebrafish were raised and maintained as described [ 8]. Ekk wild-type fish 1 y of age were obtained from EkkWill Waterlife resources (Gibsonton, Florida, United States) and Scientific Hatcheries (Huntington Beach, California, United States) and used for heart amputation according to the described procedure [ 8]. Sham-operated and heart amputated fish were allowed to regenerate for 3, 7, and 14 d in water at room temperature. Fish were then anesthetized and hearts were removed for analysis. For testing the effects of AG1295 on heart regeneration, Ekk wild-type fish were amputated as described and allowed to recover for 2 d. The fish were then kept in a 1-L tank (with 0.5 L of water) supplied with air on the bench. AG1295 (30 μM) was dissolved in DMSO and added into the fish water, which was changed every day. Control fish were treated with DMSO only or the MMP inhibitor GM6001 (10 μM) (Chemicon International, Temecula, California, United States). The fish were kept in the dark because AG1295 is light sensitive.

### Tissue collection and RNA extraction.

The heart regeneration process occurs along the amputation plane. To enrich transcripts that are involved in regeneration, we roughly dissected the one third of the ventricle containing the amputation plane as heart regenerates. Total RNA was extracted from ten to 12 sham-operated and regenerating hearts. After homogenization, total RNA was extracted using TRIzol reagent (Invitrogen, Carlsbad, California, United States), according to the manufacturer's recommendations. The quantity and quality of total RNA were assessed by absorbance at 260 nm and 280 nm and by gel electrophoresis.

### Microarray analysis.

Independent hybridizations of three biological replicates were performed for each time point. Approximately 1 μg of total RNA was converted to biotin-labeled cRNA using Gene Chip One-Cycle Target labeling kit and hybridized to the zebrafish genome array according to the manufacturer's guidelines (Affymetrix, Santa Clara, California, United States). Differentially expressed genes were primarily identified using methods implemented in Bioconductor's “affy” package and available custom scripts [ 41]. Genes that were differentially expressed in at least one time point were identified by a q value of less than 0.15 (3 dpa, 7 dpa, 14 dpa versus uncut), fold change of greater than 2, and Microarray Suite (MAS) 5.0 absent-present call. The differentially expressed probe sets were annotated based on UniGene ID. The differentially expressed genes were clustered first using the self-organizing map (SOM) followed by hierarchical cluster analysis with centroid linkage. Clustering results were visualized using JavaTreeview ( http://jtreeview.sourceforge.net/manual.html) [ 42].

### Semiquantitative RT-PCR.

cDNA was synthesized using Superscript III reverse transcriptase (Invitrogen). PCR was performed as described [ 35] using the following primer pairs (F for forward primer; R for reverse primer): *apoEb*: F 5′- AGGAGACAGCCGAGAACCTAC −3′, R 5′- ACTAATTATTTTGAATGACGC- 3′; *thymosin beta-4*: F 5′- CAGCCACTTCTGTGCCCTACG-3′, R 5′- AGCCCCAGTGACTCCAATC-3′; *cxcl12a*-1: F 5′- CGGAATTCTTCCACAGTCAACACAGT-3′, R 5′- CGGAATTCCGTCAATGCTGATAAAGC-3′; *granulin A*: F 5′- GAAGTTGTTCTTCTCCAAAAAG-3′, R 5′- TAAAGACCAAGGGAAATTAAAA-3′; *midkine*: F 5′- CGGGGTATAAAAGTAGCA-3′, R 5′- GAAGGGCAAAGTCAACGATT-3′; *vegfc*: F 5′- AGCGGGTAGTGTGGATGAAC-3′, R 5′- AGCTTATGGTGACCGGTTTG-3′; *pdgf-b*: F 5′- GGACCCTCTTCCTCCATCTC −3′, R 5′- TGGGACACGTAACTGACAGC-3′; *pdgf receptor alpha*: F 5′- ACCGAATGACCAAACCTGAG-3′, R 5′- CAGGTCTCCTGAGTCCAAGC-3′; β-actin: F 5′- TTCACCACCACAGCCGAAAGA-3′, R 5′- TACCGCAAGATTCCATACCCA-3 .

### In situ hybridization.

RT-PCR fragments (except for *thymosin* β *4*) were subcloned into pGEM-T-EZ for sequencing and for generating probes for in situ hybridization. An EST clone (IMAGE clone ID: 4967321) of *thymosin* β *4* was used to generate a probe for in situ hybridization. Digoxigenin-labeled RNA probes were transcribed using T7 and SP6 RNA polymerase (Invitrogen). Automated ISH was performed on the Discovery System (Ventana Medical Systems, Tucson, Arizona, United States). After deparaffinization, slides were fixed in 4% paraformaldehyde for 10 min and digested with proteinase K (10 μg/ml) for 10 min at 37 °C. Digoxigenin-labeled riboprobes were diluted at 1:200 in hybridization solution (RyboHybe, Ventana Medical Systems, Tucson, Arizona, United States). The hybridization was performed for 6 h at 65 °C. After hybridization, slides were washed twice in 0.1× SSC at 75 °C for 6 min. Detection was done using biotinylated anti-digoxigenin antibody (Biogenex, San Ramon, California, United States) followed by streptavidin-alkaline-phosphatase conjugate and visualized by NBT/BCIP substrate reaction (Ventana BlueMap Detection Kit). Slides were counterstained by nuclear fast red (Vector Laboratories, Burlingame, California, United States) and dehydrated for microscopic analysis. Radioactive ISH was performed using ^35^S-labeled in vitro transcribed riboprobes (Roche, Basel, Switzerland). The riboprobes were diluted in hybridization buffer at 1 × 10 ^6^ CPM per slide. Hybridization was performed overnight at 60 °C. After hybridization, slides were washed for 2 h in 0.1× SSC at 65 °C. Slides were dipped in Kodak NTB emulsion, exposed at 4 °C for 4 wk, then developed and H&E counterstained.

### Cardiomyocyte preparation and growth factor treatment.

Adult zebrafish cardiomyocytes were prepared based on the protocol by Warren et al. [ 25] and modified as follows. Fish were anesthetized in tricaine, and whole hearts were dissected out and placed in Hanks' solution. The atrium and aorta were carefully removed in Hanks' solution, and the remaining ventricle were cut into three or four small pieces to get rid of the blood and then transferred into solution A [ 25]. Another equal volume of solution A containing 2 mg/ml collagenase I (Worthington, Lakewood, New Jersey, United States) and 0.28 mg/ml protease XIV (Sigma, St. Louis, Missouri, United States) was added to the solution containing tissues and was digested by stirring at room temperature for 1 h. The cells were then pelleted by spinning at 2,000 rpm for 2 min and resuspended in L15 medium. The cells were pelleted again and resuspended in L15 medium containing penicillin/streptomycin, 25 mM HEPES, and 20 U/ml insulin. To test the effect of secreted factors, the cells were treated every 3 d with medium containing the following factors: apoE4 (Invitrogen, 10 μg/ml), *midkine* (R & D Systems, Minneapolis, Minnesota, United States, 100 ng/ml), PDGF-BB (Sigma, 100 ng/ml), PDGF-AB (Sigma, 100 ng/ml), PDGF-AA (100 ng/ml), recombinant zPDGF-BB (amino acids 79 to 187, 100 ng/ml, produced by ProMab Biotechnologies, St. Albany, California, United States), or progranulin (10 nM) [ 14]. BrdU (10 mM) was added to fresh medium every 3 d.

### In vivo BrdU labeling and quantification.

BrdU was administrated by intraperitoneal injection as described [ 8]. The three largest sections of each heart were used for BrdU counting. Seventy-five MEF2-positive nuclei along the amputation plane were counted to determine the percentage of MEF2 and BrdU double-positive cells.

## Supporting Information

Dataset S1 List of Genes Differentially Expressed During Zebrafish Heart Regeneration Affymetrix probe ID, gene symbol, gene title, functional class, and description are provided for the 662 transcripts that were differentially expressed during zebrafish heart regeneration. The results of our analysis using R include mean signal, mean fc (fold change), and q-value and are provided for each sample. After hierarchical clustering, differentially expressed genes are grouped into six clusters (I to VI; see Figure 2). The cluster assignment for each transcript is provided.
(453 KB XLS)

Figure S1 Expression Patterns of Representative Genes in Each Cluster The differentially expressed genes were grouped into six clusters (I to VI). Each gene cluster is shown separately for illustrative purposes and the representative genes are indicated graphically by plotting dpa versus log _2_ (fold change). Cluster I (genes downregulated during heart regeneration) includes *mef2a (myocyte enhancer factor 2a), pdcd/aif (programmed cell death 8/apoptosis inducing factor), tsc1 (tuberous sclerosis 1),* and *hsp90 (heat shock protein 90).* Cluster II (genes upregulated at 3 dpa) includes *apo-ci (apolipoprotein c-I), hspa5 (heat shock protein 70–5),* and *mps1 (monopolar spindle 1).* Cluster III (upregulated at 3 and 7dpa) includes *granulin A, survivin,* and *apoeb (apolipoprotein Eb).* Cluster IV (genes with highest expression at 7dpa) includes *pdgf-a (platelet-derived growth factor-a), sparc (secreted acidic cysteine–rich glycoprotein), bnpc (brain natriuretic peptide type C),* and *ctgf (connective tissue growth factor).* Cluster V (genes upregulated at all three time points) includes *fn1(fibronectin 1), mdka (midkine-related growth factor a), thymosin (thymosin* β *4), vegfc (vascular endothelial growth factor c),* and *igf2 (insulin-like growth factor 2).* Cluster VI (genes upregulated at 7 and 14 dpa) includes *mmp14b (matrix metalloproteinase 14b), cxcl12a [chemokine (c-x-c motif) ligand 12a], tbx18 (T box-18),* and *atf3 (activating transcription factor 3).*
(607 KB EPS)

Figure S2 Sequence Alignment of Human, Mouse, *Xenopus,* and Zebrafish PDGF-B
The alignments were performed using clustW. The gray boxes indicate homologous regions. The red boxes indicate conserved cysteine residues. Zebrafish PDGF-B shows high homology to other species.
(404 KB EPS)

Table S1 List of Genes Differentially Expressed during Both Heart and Fin Regeneration (44 KB DOC)

Table S2 Secreted Factors Tested on Zebrafish Cardiomyocytes for Induction of DNA Synthesis (27 KB DOC)

### Accession Numbers

The GenBank ( http://www.ncbi.nlm.nih.gov/Genbank) accession numbers for human, mouse, and *Xenopus* PDGF-B used in Figure S2 are NP_002559, NP_035187, and AAH77559. The GenBank accession numbers used in this paper are zebrafish *monopolar spindle (mps) 1* gene (NM_175042), heat shock protein 60 (hsp60) chaperone protein (NM_181330), *pdgf-a* (AF200950), *pdgf-b* (DQ647924), *apoEb* (NM_131098) *, vegf-c* (AF466147), *granulin-A* (AF375477), MMP2 (NM_198067), MMP14a (NM_194416), MMP14b (NM_194414), acidic cysteine–rich glycoprotein *(sparc)* (BG305371), *thymosin* β *4* (BI428446) *, insulin-like growth factor 2 (igf2)* (AF250289), *chemokine c-x-c ligand 12 (cxcl12)* (NM_178307), and *midkine a* (NM_131070).

## Abbreviations

dpa: days postamputation
MMP: matrix metalloproteinase
PDGF: platelet-derived growth factor
zPDGF-BB: zebrafish PDGF-BB protein
