# Supplementary material for: Gene Expression Analysis of Zebrafish Heart Regeneration
Source: PLoS Biol. 2006 Aug 1;4(8):e260. doi: 10.1371/journal.pbio.0040260 (PMC1523227; doi:10.1371/journal.pbio.0040260)
Supplement: Supplementary file 1 [file pbio.0040260.st001.doc]

Table S1. List of genes that are differentially expressed in both regenerating fin and heart

# Upregulated genes

**Functional category	Gene symbol	Gene title	Accession No.**

**Apoptosis related**	birc5a	survivin1	AY057057.1

**Apoptosis/phagocytosis**	elmo1	engulfment and cell	BI877952

motility 1

**Cell cycle**	mps1	monopolar spindle 1	NM_175042.2

cdc20	cell division cycle 20	AW116246

homolog

cdca8l	cell division cycle	BI704246

associated 8-like

plk1	polo-like kinase 1	AW116681

cdc2	cell division cycle 2	AF268044.1

ccna2	cyclin A2	NM_152949.1

zgc:112133	similar to mitotic spindle	BI866657

assembly checkpoint

protein MAD2A (MAD2-like 1)

**Cell matrix and cell**	timp2l	tissue inhibitor of	AW420494

**adhesion**	matrix matelloproteinase 2

mmp14a	matrix matelloproteinase 14	AA606011

alpha	BI475931

timpl	tissue inhibitor of metallo-	BQ131818

proteinase 2, like

mmp14b	matrix metalloproteinase 14,	BE200676

beta

LOC565402	similar to collagen type XI,	AL724939

alpha 1

col1a1	collagen type 1, alpha 1	NM_199214

BE201886

col1a2	collagen type 1, alpha 2	AI331605

BI707807

wu:fa99f12	collagen alpha I (XII)	BQ449552

nid2	nidogen 2	AI601403

fn1	fibronectin 1	NM_131520.1

AF081127.1

fn1b	fibronectin 1b	BI707688

dcn	decorin	BC044206

tgfbi	TGF beta-induced	AB086407

bgl3	biglycan-like protein 3	BQ092573

**Coagulation**	LOC561287	embryonic	BQ285074

transglutaminase

**Electron transport**	zgc:92903	thioredoxin	BI864002

pdia4	protein disulfide isomerase	BC045862.1

A4 precursor

**Inflammation**	tsg-6	TNF-a induced protein 6	BQ615550

BQ092008

tnc	tenascin C	X89203.1

**Ion binding**	fth1	ferritin heavy chain	AW232676

**Ligand/ Secreted**	LOC570224	C type natriuretic	BQ092110

Peptide 4

mdka	midkine related factor a	CD014404

igf2	insulin-like growth factor 2	AF250289

precursor

clu	clusterin	AW077466

LOC570373/	similar to apolipoprotein C-I	BM186239

LOC570638

zgc:56225/metrnl	meteorin, glial cell	BC046006.1

differentiation regulator like

**Metabolism**	AW510294	AW510294	AW510294

ugdh	UDP-glucose dehydrogenase	BM096064

zgc:110691	glucosamine-6-phosphate	BQ262910

isomerase

zgc:110343	peroxiredoxin 1	CD594679

ndpkz3	nucleoside diphosphate	NM_130928.1

kinase-Z3

cpn1	carboxypeptidase N,	BI704530

polypeptide 1

zgc:92215	histidyl-tRNA synthetase	AI957786

zgc:77447	protein-lysine 6-oxidase	BG302899

aldh1a2	aldehyde dehydrogenase 1	NM_131850.1

family, membrane A2

dut	dUTP pyrophosphatase	BQ617623

wu:fc12b04	protein-glutamine gamma-	AF242545.1

glutamyltransferase

**Nuclear import**	kpna2	karyopherin alpha 2	BI878593

zgc:101013	nuclear transport factor 2	BM402118

**Protein folding**	calrl	calreticulin like	BC046906.1

calr	calreticulin	NM_131047.1

hspa5	heat shock 70 KDa protein 5	BC052971.1

fkbp10	FK506 binding protein 10	BC045938.1

LOC570980	similar to FK506 binding	AI974136

protein 14

zgc:101826	similar to FK506 binding	BI979906

protein 2

LOC558042	peptidylprolyl isomerase-	AL717190

ike 1

**Protein transport**	kdelr3	KDEL ER protein retention	CD605145

Retention receptor 3

rrbp1	ribosome binding protein 1	AW342746

homolog

**Selenium binding**	sepm	selenoprotein M	NM_178286.2

**Signaling**	marcks	myristoylated alanine rich	BQ078442

protein kinase C substrate

LOC562094	similar to transmembrane	AW128322

phosphatase with tensin

homology

**Structural**	LOC573216	tubulin alpha 2, isoform 2	BG306212

tubb5	tubulin beta 5	AF528096.1

**Structural/transcriptional**	csrp1	cysteine and glycine-rich	BM534006

**Regulation**	protein 1

**Transcription**	phf5a	PHD finger protein 5A	NM_173266.1

atf3	activating transcription	BC045371.1

factor 3

lmo4l	LIM domain only 4, like	AF398515.1

junb	jun B proto-oncogene	BC053234.1

**Transporter**	slc39a7	solute carrier family 39	AI437101

BQ480733

zgc:56419	similar to SERP1	BC049018.1

**Unknown**	Dr.Affx.2.1.S1_at	Dr.Affx.2.1.S1_at	AY380819

id:ibd1087	id:ibd1087	BG985449

wu:fi13a08	wu:fi13a08	AW116248

LOC558965	similar to ubquitin-like	BQ075086

protein 1

wu:fd57e01	wu:fd57e01	AL720987

zgc:77059	zgc:77059	AW421190

sb:cb185	similar to cystatin B	BM184265

LOC562013	similar to MGC78892 protein	BQ284889

im:7151414	im:7151414	BM342191

zgc:63590	zgc:63590	BM181793

wu:fj86h11	similar to ubiquitin-	BM531174

conjugating enzyme E2T

LOC562022 /	similar to Olfactomedin-like	BQ074534

LOC563065 /	protein 3

LOC566649

LOC561086	similar to CG14434-PA	CD593876

zgc:103578	zgc:103578	AW281699

sb:cb377	sb:cb377	BE693156

id:ibd5063	id:ibd5063	BG985448

wu:fk30a05	wu:fk30a05	AW566977

BC046015.1	BC046015.1	BC046015.1

LOC562399	similar to ectonucleotide	BE605993

Pyrophosphatase

zgc:109978	zgc:109978	AL730317

wu:fc59b06	wu:fc59b06	AI878469

BM532441	BM532441	BM532441

zgc:77153	zgc:77153	BI877516

LOC562094	similar to transmembrane	AW128322

phosphatase with tensin

homology

AW420565	AW420565	AW420565

zgc:77327	zgc:77327	zgc:77327

wu:fi13a08	wu:fi13a08	AW116248

wu:fc60b09	wu:fc60b09	AL715802

wu:fb09f08	wu:fb09f08	AI397304

CB362946	CB362946	CB362946

wu:fc87g11	wu:fc87g11	AI943264

AL913978	AL913978	AL913978

wu:fl17h04	wu:fl17h04	BE558041

wu:fl03b09	wu:fl03b09	BE201936

wu:fa99c11	wu:fa99c11	AI942877

id:ibd5063	id:ibd5063	BG985448

AI545309	AI545309	AI545309

wu:fj65f09	wu:fj65f09	BQ092092

LOC553997	progranulin-1progranulin-2	AY289607.1

antisense transcript

zgc:86646	zgc:86646	AI793417

zgc:91863	zgc:91863	BI428259

zgc:86839	zgc:86839	AI959618

zgc:100902	zgc:100902	AI943251

zgc:103420	zgc:103420	zgc:103420

# Downregulated genes

**Functional category**	Gene symbol	Gene title	Accession No.

**Calcium binding**	anxa4	annexin 4	AW117084

**Metabolism**	arg2	arginase type II	BG891983

**Ligand/Secreted**	inhbb	inhibin beta B	NM_131068.1

**Signaling**	zgc:55498	similar to mitogen-activated	BC044411.1

protein kinase 4 (ERK4)

**Transporter (plasma)**	rbp4	retinol binding protein 4	AW232202

**Transcription**	reverbb1	rev erb beta 1	BF937474

klf12	Kruppel-like factor 12	NM_131859.1

BI878775	similar to basic transcription	BI878775

element binding protein 1

**Unknown**	wu:fa94b01	wu:fa94b01	AI332232

zgc:110777	zgc:110777	BG305771

wu:fc63h12	wu:fc63h12	AI878665

wu:fb81c07	wu:fb81c07	CD283215

LOC572832	putative nucleic acid binding	BI864314

protein

# Upregulated in heart but downregulated in fin

**Metabolism**	zgc:65956	zgc:65956	AI883922

**Immune response**	zgc:92049	MHC clasII integral	NM_131695.1

membrane protein alpha

chain 3

**Unknown**	BG884598	BG884598	BG884598

LOC566658	LOC566658	BE201834

zgc:63831/	zgc:63831/LOC554451	AI332262

LOC554451

wu:fc33f12	wu:fc33f12	AI723186

AW279710	AW279710	AW279710

CD283908	CD283908	CD283908

# Downregulated in heart but upregulated in fin

**Protein folding**	hsp90a	heat shock protein-90 alpha	BM103343

**Structural**	kif11	kinesin family member 11	NM_173261.1

**Transcription**	cited3	similar to Cbp/p300-	BG305277

interacting transactivator,

cited3
