# Supplementary material for: Gene Expression Analysis of Zebrafish Heart Regeneration
Source: PLoS Biol. 2006 Aug 1;4(8):e260. doi: 10.1371/journal.pbio.0040260 (PMC1523227; doi:10.1371/journal.pbio.0040260)
Supplement: Supplementary file 2 [file pbio.0040260.st002.doc]

Table S2. Induction of DNA synthesis in zebrafish cardiomyocytes by secreted factors

| Factor tested on zebrafish cardiomyocytes | Fold increase of DNA synthesis in  cardiomyocytes |
| --- | --- |
| ApoE4 | 0.76 ± 0.03 |
| Granulin | 1.15 ± 0.05 |
| midkine | 1.01 ± 0.03 |
| PDGF-AA | 0.95 ± 0.04 |
| PDGF-AB | 1.27 ± 0.06 |
| PDGF-BB | 2.68 ± 0.05* |

* P<0.05
